# The Influence of Heredity on Disease, with Special Reference to Tuberculosis, Cancer, and Diseases of the Nervous System. A Discussion

**Published:** 1909-12

**Authors:** 


**The Influence of Heredity on Disease, with special reference to Tuberculosis, Cancer, and Diseases of the Nervous System. A Discussion.** Pp. xii, 142. London: Longmans, Green and Co. 1909.

A survey of this discussion on heredity in disease, with special reference to cancer, tubercle, and insanity, from the *Proceedings of the Royal Society of Medicine,* cannot fail to impress upon the reader the exceptional difficulties which have yet to be overcome before the whole question can be placed upon a useful scientific basis.

Dr. G. H. Savage, Dr. F. W. Mott, and Dr. Mercier point out that with mental cases it is almost impossible to obtain reliable statistics.

Dr. Mercier goes so far as to say, "The compilation of the statistics of inheritance which appear year after year in the Reports of the Commissioners in Lunacy is a gigantic waste of time and labour." Dr. Mott, however, has been able to relate some really remarkable family histories, and Dr. Mercier states that in some cases which have come under his notice the insane members in the families have been distributed approximately in accordance with Mendelian rules.

A family history of pseudo-hypertrophic paralysis quoted by Sir W. Gowers affords a good illustration of Mendelian segregation.

The discussion on tuberculosis was opened by Dr. Latham, who weighs very carefully the various circumstances affecting the increase and the check of this disease. Dr. Latham quotes the tuberculosis statistics of New York to illustrate the results obtainable by advanced sanitation, and, arguing from the fact that in autopsies a very large proportion of adult bodies may be shown to have at some time suffered from one or other of the many forms of tuberculosis, he concludes that the communities concerned have acquired a degree of immunity.

It is surprising to find a man of Professor Pearson's standing referring later to this racial acquired immunity as an example of the inheritance of acquired features.

Dr. Latham holds the now generally-accepted view that the theory of inherited predisposition to tuberculosis is based on insufficient evidence, the real factors at work being the surroundings and the sources of infection to which those children born of tuberculous parents are exposed.

Sir J. McFadyean, who held a brief for the lower animals, lays special stress on the question of bovine tuberculosis being the result of exposure to infection and not of predisposition. In support of this statement he mentions that Jersey cattle appear to be immune in Jersey because the disease has not yet invaded their island, but when reared in England amidst tuberculous surroundings they show no special resistance to infection.

In the matter of heredity in cancer, Dr. Bashford and Mr. Butlin hold opposite views. Dr. Bashford, depending chiefly upon statistics and laboratory evidence, says : " We have not yet obtained even an indication that cancer is inherited." Mr. Butlin, on the other hand, deriving his impressions from his clinical experience, is of the opinion that a tendency to the disease is heritable, and in making his point he applies adverse criticism to the arguments of Dr. Bashford, who, he says, " would exclude those families in which the disease has skipped a generation, and those families in which the theory of inheritance depends largely on cancer in collateral members, and those families in which the disease is not of the same variety in all the cancerous members," thus leaving no evidence by which to establish the theory of heredity.

As might be expected, these discussions did not lead to peace being declared between the Mendelian and the Biometrician camps ; in fact, on December 2nd, at the last meeting of the series, a strenuous attack on Professor Pearson's methods was made by Mr. G. P. Mudge. It is difficult in this attack, and the reply to it, to trace any sign of pending reconciliation between the two schools.

The book should be read and re-read.

